# Radioimmunotherapy in colorectal cancer treatment: present and future

**DOI:** 10.3389/fimmu.2023.1105180

**Published:** 2023-05-10

**Authors:** Jingyi Shi, Zhuang Sun, Zhaoya Gao, Dandan Huang, Haopeng Hong, Jin Gu

**Affiliations:** ^1^ Key Laboratory of Carcinogenesis and Translational Research (Ministry of Education/Beijing), Department of Gastrointestinal Surgery III, Peking University Cancer Hospital & Institute, Beijing, China; ^2^ Department of Gastrointestinal Surgery, Peking University Shougang Hospital, Beijing, China; ^3^ Department of General Surgery, Peking University First Hospital, Beijing, China; ^4^ Department of Oncology, Peking University Shougang Hospital, Beijing, China; ^5^ Peking Tsinghua Center for Life Science, Peking University International Cancer Center, Beijing, China

**Keywords:** colorectal cancer, radiotherapy, immunotherapy, metastasis, locally advanced rectal cancer

## Abstract

Colorectal cancer (CRC) is a deadly form of cancer worldwide. Patients with locally advanced rectal cancer and metastatic CRC have a poor long-term prognosis, and rational and effective treatment remains a major challenge. Common treatments include multi-modal combinations of surgery, radiotherapy, and chemotherapy; however, recurrence and metastasis rates remain high. The combination of radiotherapy and immunotherapy (radioimmunotherapy [RIT]) may offer new solutions to this problem, but its prospects remain uncertain. This review aimed to summarize the current applications of radiotherapy and immunotherapy, elaborate on the underlying mechanisms, and systematically review the preliminary results of RIT-related clinical trials for CRC. Studies have identified several key predictors of RIT efficacy. Summarily, rational RIT regimens can improve the outcomes of some patients with CRC, but current study designs have limitations. Further studies on RIT should focus on including larger sample sizes and optimizing the combination therapy regimen based on underlying influencing factors.

## 1 Introduction

As the third most common cancer (10%) and the second most lethal cancer (9.4%) worldwide (1), colorectal cancer (CRC) is a disease that threatens the health of the populace and poses a major economic burden. Improving the prognosis of patients with locally advanced rectal cancer (LARC) and metastatic CRC (mCRC) remains an important and challenging problem. Patients with mCRC have poor prognoses, with a 5-year survival rate of less than 15% (2). In the past few decades, radiotherapy has made great progress in controlling local tumor progression with reduced side effects; however, controlling distant lesions outside the irradiated field remains a major challenge. In recent years, immunotherapy has become the fourth pillar of cancer treatment in addition to surgical therapy, chemotherapy, and radiotherapy, and is increasingly recognized as a promising and attractive partner for radiotherapy. Compared to traditional chemotherapy, immunotherapy has higher specificity, does not affect normal cells without tumor-specific antigens, and can synergize with the abscopal effect of radiotherapy (3). In recent clinical trials of melanoma, non-small cell lung cancer, and renal cell carcinoma, RIT effectively controlled tumor progression (4). Several case reports and preclinical studies have shown a promising effect of RIT for patients with CRC; however, specific protocols are required to determine treatment dosage, target selection, course of treatment, and immunotherapy timing. Many clinical trials have been registered and initiated, the data of which are expected to guide and optimize the RIT regimen, clarifying its role in CRC treatment. Here, we aimed to review the studies related to RIT for patients with CRC, summarized the current results of clinical trials, and make prospects for its future practical application.

## 2 Current roles of radiotherapy and immunotherapy in treating patients with CRC

### 2.1 Radiotherapy in CRC

The definition of LARC differs as stage II (cT3-4, N0) or Stage III (any cT, N+). Neoadjuvant radiotherapy combined with concurrent chemotherapy is currently the standard preoperative treatment for stages II-III rectal cancer (5). For patients with LARC, preoperative radiotherapy is more effective at reducing tumor volume, increasing the likelihood of anal sphincter preservation, and reducing local recurrence. However, radiotherapy can also increase toxic reactions (such as radiation damage and hematological toxicity) (6). Neoadjuvant radiotherapy is generally divided into long-course radiotherapy (50.4Gy, 25–28 fractions) and short-course radiotherapy (25Gy, 5 fractions). Patients in the long-course group were more likely to develop serious complications, such as radiation dermatitis, than those in the short-course group (0% vs. 5.6%, *P* =0.003). However, the short-course group had a higher likelihood of developing permanent postoperative stoma than the long-course group (38.0% vs. 29.8%, *P* = 0.13) (7). The rates of R0 resection (87% vs. 90%, *P* = 0.554), pathological complete response (12% vs. 10%, *P* = 0.740), and overall tumor downstaging (75% vs. 75%, *P* = 0.920) were similar between these two treatments (8). Therefore, the optimal course of preoperative radiotherapy for patients with LARC remains. Total neoadjuvant therapy (TNT) has gradually gained attention as a preferred neoadjuvant therapy scheme. TNT usually advocates neoadjuvant radiotherapy or chemotherapy combined with radiotherapy, chemotherapy, or immunotherapy, to reduce the rate of total mesorectal excision (TME) surgery and achieve a higher rate of pathological complete response (pCR) (9). Some studies have shown that TNT improves the overall pCR rate, DFS, and overall survival (OS) and reduces the risk of distant metastasis compared to standard treatment (10). However, the current TNT regimen has not reached optimal results. Moreover, defining the role of radiotherapy and the choice between chemotherapy and immunotherapy is still under discussion.

Radiotherapy has also been used in treating patients with mCRC. Colorectal cancer liver metastasis (CRLM) is the most common distant metastasis. Approximately 15%-25% of patients develop CRLM during the diagnosis of primary cancer (synchronous metastases), and 15%-25% of patients develop CRLM within 5 years after the first diagnosis (metachronous metastases) (11). Stereotactic ablative radiotherapy (SABR) is a concentrated and highly-precisive form of radiotherapy that can improve the survival rate of patients with CRLM, especially for those who are not eligible for surgery. However, the recurrence rate of CRLM after SABR still remains 5.3%–29% in the treatment field and 59% outside, making it necessary to combine SABR with other systemic therapies (12). The limited application of SABR could also be attributed to the tolerated dose of normal liver tissues being much lower than the lethal dose to tumor cells. Lung metastasis is the second most common distant metastasis after CRLM, with nearly 30% of patients developing metastasis throughout CRC (13). For those inoperable CRC lung metastases, SABR is also a practical choice (14).

### 2.2 Immunotherapy in CRC

Compared with chemotherapy and radiotherapy, immunotherapy mainly utilizes the patient’s immune system to fight cancer cells (15, 16). Cancer immunotherapy targets specific antigens on cancer cells, alerting the immune system and coordinating the immune response to eradicate cancer, leaving normal cells without cancer cell antigens unaffected. Cancer immunotherapy can be classified based on the different immune mechanisms involved, including passive immune mechanism, active immune mechanism, or antigen-specific immune responses. Passive immunotherapy includes tumor-targeting monoclonal antibodies, adoptive cells, and oncolytic virus therapy. Active immunotherapy includes the immunomodulatory monoclonal antibodies, anti-cancer vaccines, immunostimulatory cytokines, immunosuppressive metabolic inhibitors, pattern recognition receptor agonists, and inducers of immunogenic cell death (17). Immune checkpoints (IC) are key regulators of immune reactions and act as brakes for overaction. However, the overexpression of IC contributes to immunosuppression and facilitates the proliferation and spread of malignant cells. Immune checkpoint inhibitors (ICIs) can restore immune function by targeting or blocking immune checkpoint protein ligands on the surface of T cells or other immune cell subsets. Currently, clinically approved agents are restricted to those targeting programmed cell death 1 (PD-1)/programmed cell death-ligand 1(PD-L1) or cytotoxic T-lymphocyte antigen 4 (CTLA4), based on various immunotherapy-related preclinical and clinical studies for CRC (18).

Results from KEYNOTE016 (NCT01876511) first revealed that 28 patients with deficient mismatch repair high microsatellite instability (dMMR-MSI-H) CRC receiving pembrolizumab (anti-PD-1 antibody) had a response rate (RR) of 50% (95% CI 31–69%) and disease control rate (DCR) of 89% (19). These promising results officially triggered an increase in the exploration of CRC immunotherapy, and many clinical trials have since been registered. One of the most promising results from ASCO 2022 presented a prospective, single-arm, Phase II study investigating the efficacy of neoadjuvant dostarlimab (anti-PD-1) in dMMR LARC (20). Thirteen patients with stage II and III dMMR rectal cancer were included. Seven patients achieved a complete clinical response after induction therapy and then were placed on hold without chemotherapy or surgery, and no serious adverse events were reported. This study’s incredible 100% clinical response rate suggests new directions for immunotherapy. However, current ICIs have no significant effect on proficient mismatch repair (pMMR), microsatellite stable (MSS), or low microsatellite instability (MSI-L) tumors (known as pMMR-MSI-L tumors). Low tumor mutation burden (TMB) and lack of immune cell infiltration may be the mechanisms of immunotherapy resistance (21). Improving the outcomes of these patients using immunotherapy is a key problem.

## 3 Coeffects of radiotherapy and immunotherapy in CRC

**Figure 1:**
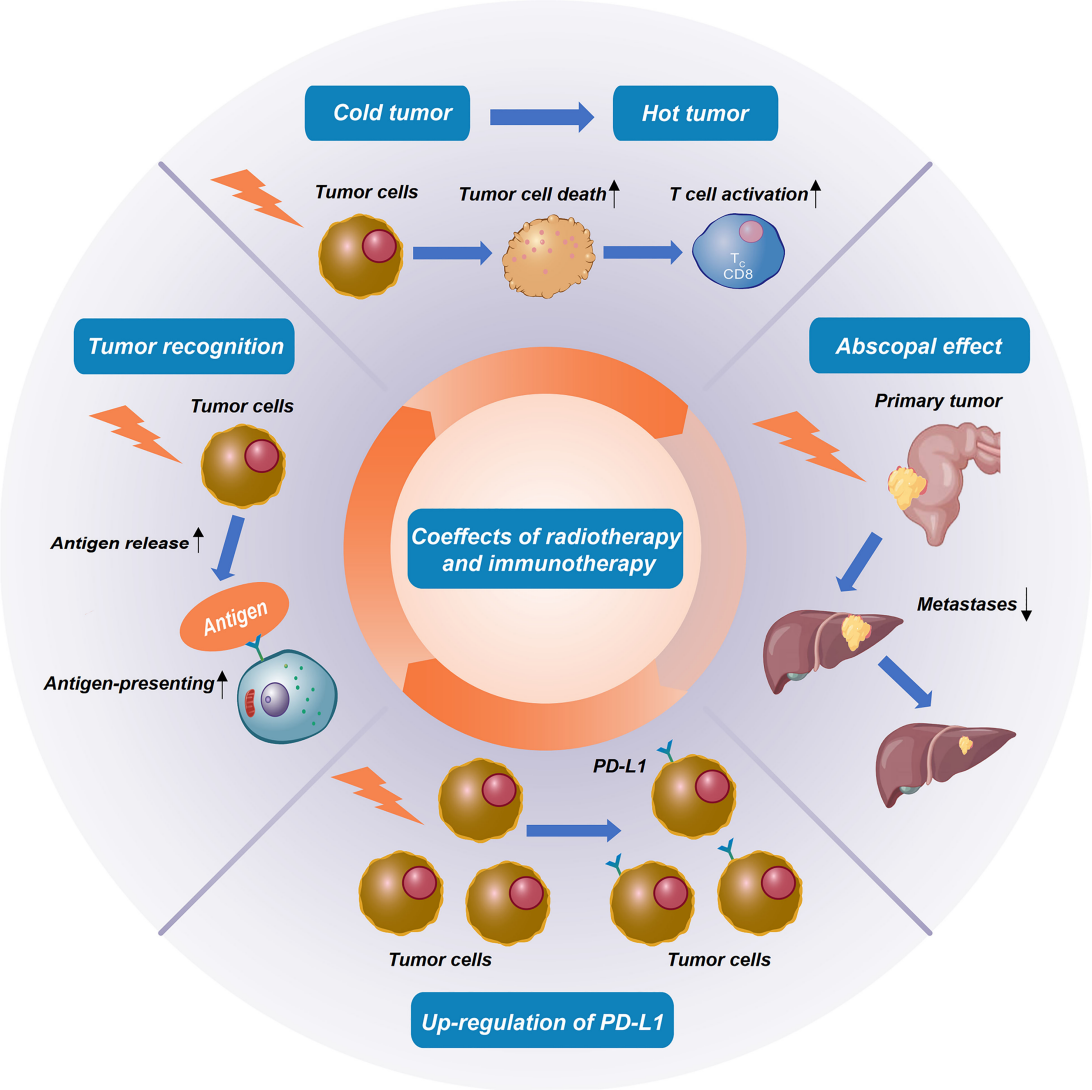
Coeffects of radiotherapy and immunotherapy in colorectal cancer.

RIT using radioactive element-labeled monoclonal antibodies has been applied to various animal models and clinical trials in patients since the emergence of hybridoma technology in 1975 and has shown effectiveness in non-Hodgkin’s lymphoma, beginning the exploration of RIT (22). RIT uses monoclonal antibodies to ship radionuclides specifically to the cancer cells, delivering high doses of therapeutic radiation to cancer cells while minimizing exposure to normal cells. Clinically, RIT is widely used in the most radiosensitive tumors such as leukemia and lymphoma. For solid tumors, direct intravenous administration of radioactive antibodies has been relatively unsuccessful (23, 24). Therefore, radiotherapy combined with immunotherapy as the new RIT regimen is more widely used in the treatment of CRC after a few immunotherapy drugs were approved. Most importantly, unique interactions between the radiological effects of radiotherapy and the immune system have various coeffects on local and systemic tumor control (**Figure 1**).

### 3.1 Radiation promotes the release and recognition of tumor-specific antigens

Radiation can enhance the clearance of damaged tumor cells by antigen-presenting cells, thereby promoting the initiation of T cells. The expression of the major histocompatibility complex class I (MHC-I) on tumor cells reduces the recognition ability of cytotoxic T cells to cancer cells. Radiation can up-regulate MHC-I on the tumor surface, promote the recognition of tumor-specific antigens by antigen-presenting cells, and enhance the killing effect of cytotoxic T cells (25).

### 3.2 Radiation turns cold tumors into hot tumors

Radiation can directly damage cancer cells, leading to their death and triggering the activation of CD8^+^ T cells, whereas changes in the immune microenvironment, involving dead tumor cells and surrounding stromal cells, can trigger systemic immune responses. Thus, the “one-two punch” of combined radiation and immunotherapy may be more effective than either treatment alone because of their unique synergies. This may turn cold tumors (not sensitive to common therapies) into hot tumors (responding better to the same therapies) (26). Due to the close interrelation between radiation and the immune system, whether immunotherapy can amplify the systemic effect of radiotherapy and turn radiotherapy into an “*in situ* vaccine”, to make “cold” tumors rejected by the immune system react to immunotherapy or make patients resistant to radiotherapy sensitive to radiation, has become a new research direction (27).

### 3.3 Radiotherapy induces the abscopal effect

Radiation can also induce T cell-mediated suppression of untreated distant tumors by inducing tumor antigen recognition (known as the abscopal effect) (28). The abscopal effect of amplified radiotherapy is significant for treating patients with mCRC and is an important complement to the current immunotherapy regimen alone. Many preclinical studies have shown that combined immunotherapy can enhance the abscopal effect of radiotherapy. CD4^+^ regulatory T cells (Tregs) are highly immunosuppressive cell subsets that, when overexpressed, can hinder immune surveillance of cancer, prevent effective anti-tumor immune responses, and promote tumor progression. Activating CTLA-4 can specifically upregulate Tregs; therefore, anti-CTLA-4 antibodies can effectively kill effector Tregs or weaken their immunosuppressive activity (29). Studies have shown that compared with radiotherapy alone, radiotherapy combined with anti-CD25/CTLA4 monoclonal antibody can significantly increase CD8+ T cell proportion and the CD8^+^/CD4^+^ratio in patients with LARC (*P <*0.05), and reduce Tregs, PD-1^+^ CD8^+^ T cells, and PD-1^+^ CD4^+^ T cells (*P <*0.05). RIT inhibited the growth of local and distal unirradiated tumors, improved the OS rate, and reduced the incidence of liver metastasis in mouse models (*P <*0.05) (30). Dewan MZ et al. (31) also found that fractionated radiotherapy combined with 9H10 (anti-CTLA-4 antibody) in two kinds of preclinical CRC models can induce the abscopal effect. SABR targeting metastases can also differentially activate the cytotoxicity of malignant cells and the cell protection pathway of non-malignant cells, resulting in significant changes in the tumor immune microenvironment. The synergistic effect of SABR and immunotherapy may enhance the abscopal effect of irradiation on field lesions by targeting different links of the immune response (12).

### 3.4 Radiation induces the up-regulation of PD-L1 on tumor cells

PD-1 is an inhibitory receptor expressed by all activated T cells, and PD-L1 shows broad expression on both hematological and non-hematological cells, making the PD-1 pathway as a key regulator of immune cell functions. Studies have shown that blocking the PD-1 pathway can promote durable antitumor immune responses (32). Local RT could induce an immunogenic antitumor response that is partially counteracted by upregulation of PD-L1 and transformation of growth factor β (TGF-β) (33). Fractional radiotherapy with αPD-1 or αPD-L1 monoclonal antibody treatment can produce an effective CD8^+^T cell response, thus improving local tumor control, long-term survival, and effectively preventing tumor recurrence.

### 3.5 Radiotherapy might affect the expression of MMR-related genes

Incheol Seo et al. (34) reported that radiation could induce downregulation of MMR system-related genes in three CRC cells. In addition, they analyzed RNA sequencing data from 60 pairs of LARC tissues before and after irradiation, and found that the MMR-related gene set was significantly downregulated in tissues after chemoradiotherapy (CRT). Notably, they also found that one patient with LARC showed a change in MSI status from MSS to MSI-low after CRT. As downregulation of MMR system-related genes and MMR deficiency could cause MSI which has been found to predict a good response to PD-1 blockade (35, 36), this effect might contribute to the combination of radiotherapy and immunotherapy in CRC.

## 4 Studies on RIT in CRC patients

**Figure 2:**
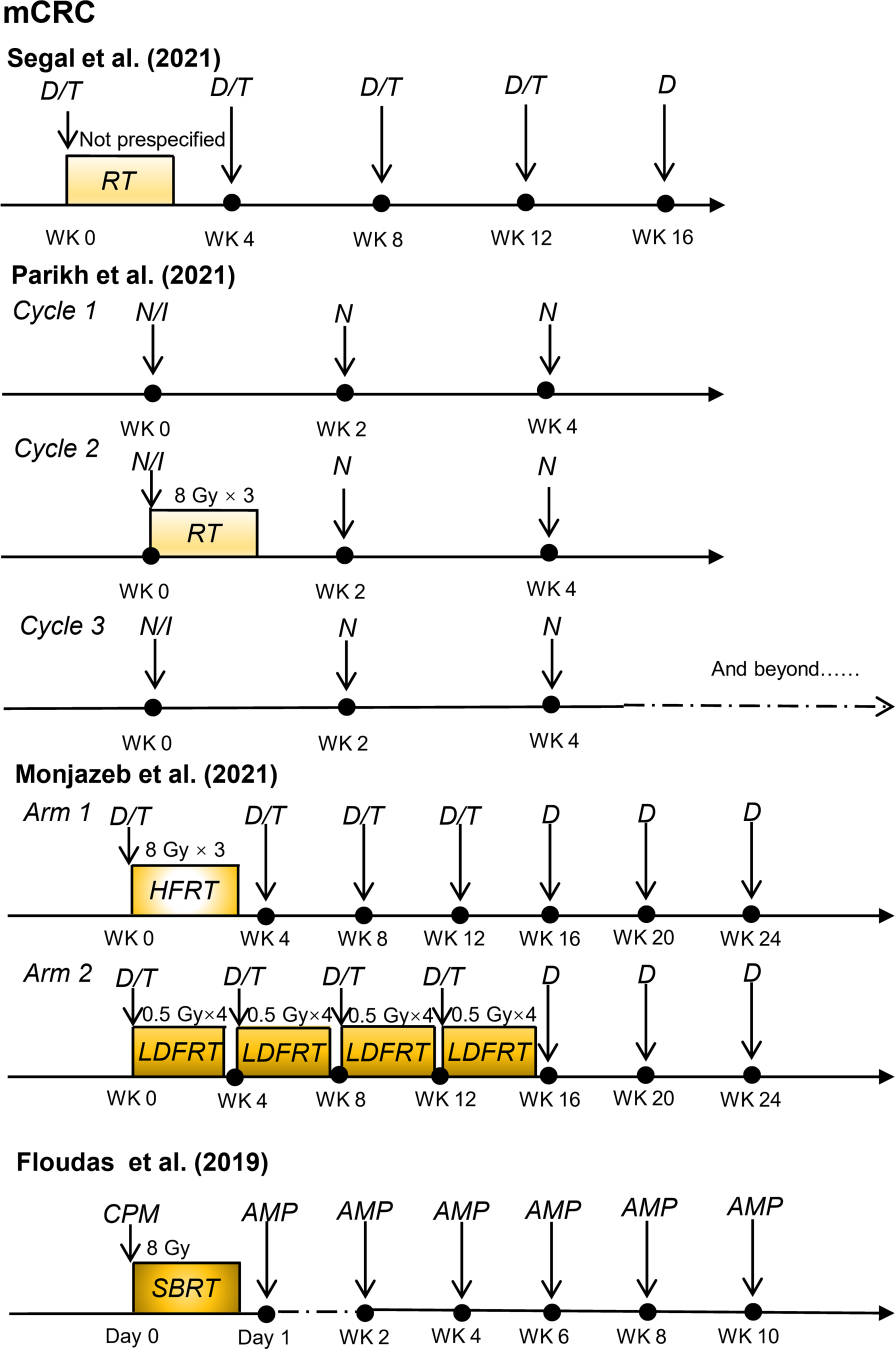
Radioimmunotherapy regimens in current clinical trials for patients with metastatic colorectal cancer. (mCRC, metastatic colorectal cancer; D, durvalumab; T, tremelimumab; RT, radiotherapy; N, nivolumab; I, ipilimumab; HFRT, hypofractionated radiation therapy; LDFRT, low-dose fractionated radiation therapy; SBRT, stereotactic body radiotherapy; CPM, cyclophosphamide; AMP, programmed cell death 1 (PD-1) targeting agent AMP-224; WK, week).

**Figure 3:**
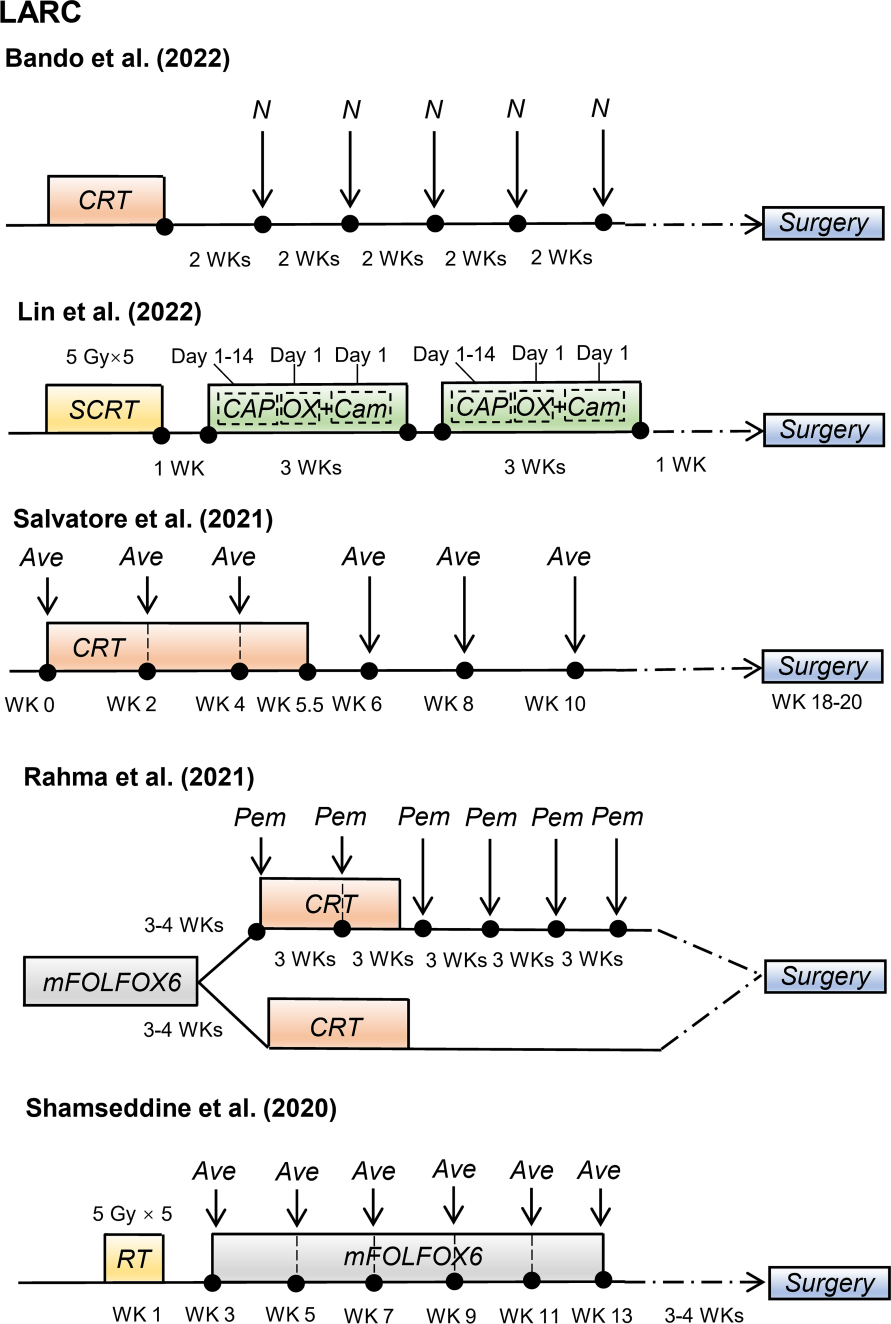
Radioimmunotherapy regimens in current clinical trials for patients with locally advanced rectal cancer. (LARC, locally advanced rectal cancer; N, nivolumab; CRT, chemoradiotherapy; SCRT, short-course radiotherapy; CAPOX, oxaliplatin plus capecitabine; Cam, camrelizumab; Ave, avelumab; mFOLFOX6, modified 5-fluorouracil (5-FU), leucovorin, oxaliplatin; Pem, pembrolizumab; WK, week).

Recently, some promising advances have been achieved in clinical studies on RIT in patients with CRC, including those with mCRC and LARC (**Table 1**). The regimens of these clinical trials for patients with mCRC and patients with LARC are shown in **Figures 2** and **3**, respectively.

**Table 1:** Clinical trials of combination of radiotherapy and immunotherapy.

Study Name (yrs.)	Study type	Participants	Treatment regimen	Number of participants	Main Outcome	ORR	Toxicity	Median PFS and OS
Floudas et al. (2019) (37)	USA single-center pilot study	Patients with mCRC refractory to standard chemotherapy	PD-1 targeting agent AMP-224 combined with low-dose cyclophosphamide and stereotactic body radiotherapy	N=15	No CR or PR. SD: three patients (20%)	0	Nine patients (60%): Grade 1 or 2. No grade 3 or 4.	PFS: 2.8 m (95% CI: 1.2–2.8 m) OS: 6.0 m(95% CI: 2.8–9.6 m)
Monjazeb et al. (2021) (38)	USA multicenter phase II study	Patients with MSS mCRC	low-dose fractionated RT (LDFRT) or hypofractionated radiation (HFRT) with PD-L1/CTLA-4 inhibition	N=18 (LDFRT: n=8. HFRT: n=10)	No CR or PR. SD: one patient	0	16 patients (84%) had toxicity, and 8 patients (42%) had grade 3–4 toxicity.	PFS: 1.7 m(90%CI: 1.5–1.8 m) OS: 3.8 m(90% CI: 2.3–5.7 m)
Segal et al. (2021) (39)	USA Phase II Single-arm Study	Patients with MSS mCRC	Durvalumab and Tremelimumab with Concurrent Radiotherapy	N=24	ORR: 8.3% (2/24) (95% CI: 1.0% to 27.0%)	ORR: 8.3%	Six patients (25%): treatment-related grade 3–4 adverse events.	PFS: 1.8 m(95% CI, 1.7–1.9) OS: 11.4 m(95% CI, 10.1–17.4)
Parikh et al. (2021) (40)	USA open-label, single-arm, non-randomized Phase 2 trial	Patients with MSS mCRC	combining radiation (8 Gy x 3), ipilimumab and nivolumab	N=40	DCR: 25% (10/40; 95% CI: 13–41%) ORR:10% (4/40; 95% CI: 3–24%)	ORR:10%	AEs related to immunotherapy Grade ≥3: 70% (53% grade 3, 15% grade 4 and 3% grade 5)	PFS: 2.4 m(95% CI: 1.8–2.5) OS: 7.1 m(95% CI: 4.3–10.9)
Shamseddine et al. (2020) (41)	Lebanon single-arm, multi-center phase II trial	Patients with LARC	short-course radiation followed by six cycles of mFOLFOX6 with avelumab	N=12	pCR: 25%(3/12) near pCR: 25%(3/12)	major pathologic response: 50%	No grade 4.	NA
Rahma et al. (2021) (42)	USA open-label, phase II, randomized clinical trial	Patients with stage II/III LARC	neoadjuvant FOLFOX plus chemoradiotherapy (capecitabine with 50.4 Gy) with or without pembrolizumab	N=185 Control arm(CA): n=95 Pembrolizumab arm(PA): n=90	neoadjuvant rectal score: CA: 11.53; PA: 14.08, *P*=0.26 pCR: CA: 29.4%; PA: 31.9%, *P*=0.75	pCR: 31.9%	grade 3-4: PA (48.2%); CA (37.3%)	NA
Salvatore et al. (2021) (43)	Italian multi-center, phase II study	Patients with resectable LARC	preoperative chemoradiotherapy plus avelumab	N=101	pCR: 23%(22/96); Major pathological response: 61.5% (59/96)	pCR: 23%	Rate of grade 3–4 non-immune and immune-related adverse events was 8% and 4%.	NA
Lin et al. (2022) (44)	China Phase II, single-center, single-arm trial	Patients with LARC (T3-4N0M0 or T1-4N+M0)	preoperative short-course radiotherapy followed by chemotherapy (CAPOX) and camrelizumab	N=27	pCR (ypT0N0) rate: 48.1% (13/27)	pCR: 48.1%	Immune-related adverse events were all grade 1–2. No grade 4/5.	NA
Bando et al. (2022) (45)	Japan single-arm phase I/II trial	Patients with MSS and MSI-H LARC	Preoperative Chemoradiotherapy plus Nivolumab before Surgery	N=44 (MSS: n=39; MSI-H: n=5)	pCR: MSS: 30% [11/37; 90%CI: 18–44%]; MSI-H: 60%(3/5)	pCR: MSS: 30%; MSI-H: 60%	Immune related severe adverse events: three patients.	NA
Carrasco et al. 2021 (46)	Belgium phase Ib/II study	patients with stage II/III RC	preoperative combination of radio-chemotherapy plus atezolizumab	N=26	pCR: 6/25 (24%)	pCR: 24%	grade 3-4: 9/26 patients	NA

mCRC, metastatic colorectal cancer; LARC, locally advanced rectal cancer; RC, rectal cancer; MSS, microsatellite stable; MSI-H, microsatellite instability high; pCR, pathologic complete response; DCR, disease control rate; ORR, objective response rate; m, months; AE, adverse event; PFS, progression-free survival; OS, overall survival; CR, complete response; PR, partial response; SD, stable disease; NA, not available.

### 4.1 Combination of radiotherapy and immunotherapy in patients with mCRC

Several clinical studies have confirmed the critical role of immunotherapy in patients with MSI-H mCRC (47, 48); however, the effect of immunotherapy in MSS mCRC remains unsatisfactory. Preclinical studies have shown that radiotherapy can increase T cell infiltration and have a synergistic effect with immunotherapy agents (31, 49). Therefore, radiotherapy might enhance the systemic response to immunotherapy in patients with MSS mCRC.

Several clinical trials have explored the safety and efficacy of radiotherapy and immunotherapy in patients with MSS mCRC. In a single-arm phase II study conducted by Neil H. Segal et al. (39), 24 patients with chemotherapy-resistant pMMR mCRC received duvarumab (anti-PD-L1 antibody) plus tremelimumab (anti-CTLA-4 antibody) and radiotherapy with a mean radiation dose of 45 Gy. After a median follow-up of 21.8 months, the objective response rate (ORR) for non-irradiated lesions was 8.3%. Overall, 25% of patients had treatment-related grade 3–4 adverse events. Increased activation of circulating CD8^+^T lymphocytes was observed in patients with an objective response, implying that radiotherapy combined with ICIs might lead to systemic immunity in pMMR/MSS mCRC. In another single-arm, non-randomized Phase II trial, Parikh et al. (40) reported that 40 patients with MSS mCRC were treated with combined radiation (8 Gy × 3), ipilimumab (anti-CTLA-4 antibody), and nivolumab (anti-PD-1 antibody), with a DCR and ORR of 25% and 10%, respectively. In a per-protocol analysis of patients who received radiotherapy (27/40), the ORR was 15%. These two early clinical trials may prove the feasibility of RIT in patients with MSS mCRC.

Monjazeb et al. (38) investigated the efficacy of durvalumab (anti-PD-L1 antibody) plus tremelimumab and radiotherapy at different radiation doses and fractions in patients with MSS mCRC (n=18). The study results demonstrated that hypofractionated radiotherapy (8 Gy × 3), or low-dose fractionated radiotherapy (0.5 Gy × 16) did not result in an objective response in the disease outside of the radiotherapy field but could change in the local immune microenvironment and increase systemic immunogenicity.

### 4.2 Combination of radiotherapy and immunotherapy in patients with LARC

Preoperative CRT can induce increased PD-L1 expression in rectal cancer (50–53), suggesting the potential benefits of combining radiotherapy or CRT with PD-1/PD-L1 blockade for an enhanced neoadjuvant strategy. Thus, clinical studies have investigated the efficacy of this combination in patients with LARC.

Salvatore et al. (43) conducted a phase II study with 101 patients with resectable LARC. These patients received standard preoperative CRT plus six cycles of avelumab (anti-PD-L1 antibody) followed by TME surgery. Of the 96 patients evaluated for pathological response, 23% (22/96) patients achieved pCR. The incidences of grades 3–4 non-immune and immune-related adverse events were 8% and 4%, respectively. Recently, Hideaki Bando et al. (45) reported that preoperative CRT followed by five cycles of nivolumab increased the pCR rate, with pCR rates of 30% (11/37) and 60% (3/5) in patients with MSS and MSI-H LARC, respectively. These clinical trials suggest that combining preoperative CRT and immunotherapy might improve the pCR rate in certain patients with LARC, with a manageable safety profile.

TNT is a novel preoperative treatment for patients with LARC that combines induction or consolidation chemotherapy with CRT before surgery (54). Lin et al. (44) reported the treatment efficacy and safety of 27 patients with LARC who received preoperative short-course radiotherapy, followed by chemotherapy (capecitabine and oxaliplatin) and camrelizumab (anti-PD-1 antibody). The pCR rate was 48.1%, including 46.2% (12/26) for MSS LARC and 100% (1/1) for dMMR LARC. All immune-related adverse events were grades 1–2. In a phase II randomized clinical trial, Rahma et al. reported that adding pembrolizumab to CRT after FOLFOX (5-fluorouracil, leucovorin, and oxaliplatin) treatment did not significantly increase the pCR rate compared to treatment with FOLFOX and CRT alone (31.9% vs. 29.4%, *P* = 0.75), although the pCR rate of adding of pembrolizumab to CRT was higher. Thus, further studies are required to prove the superiority of adding immunotherapy to TNT in patients with LARC.

Moreover, several phase II clinical trials focusing on different neoadjuvant radiotherapy regimens with other immunotherapeutic drugs in patients with LARC are ongoing (**Table 2**). The immunotherapy drugs used included durvalumab, toripalimab (anti-PD-1 antibody), tislelizumab (anti-PD-1 antibody), sintilimab (anti-PD-1 antibody), and pembrolizumab.

**Table 2:** Combination of radiotherapy and immunotherapy in clinical trials (ongoing).

Study Name (yrs.)	Study type	Participants	Treatment regimen	Clinical trials. Gov number
Hanna et al. (2021) (55)	UK multi-center, open label, phase II, randomized trial	Patients with newly diagnosed LARC	Short-course radiotherapy with concomitant durvalumab followed by FOLFOX and durvalumab or long-course chemoradiotherapy with durvalumab followed by FOLFOX and durvalumab.	NCT04621370
Wang et al. (2022) (56)	China Multi-center, double-arm, phase II trial	Patients with LARC (T3-4/N +)	short-course radiotherapy combined with chemotherapy (CAPOX) and Toripalimab	NCT04518280
Yang et al. (2022) (57)	China Multi-center, phase II trial	Patients with LARC	Long-course neoadjuvant chemoradiotherapy plus tislelizumab followed by TME surgery	NCT04911517
Li et al. (2022) (58)	China Multi-center, single-arm, phase Ib trial	Patients with MSI-H/dMMR LARC	sintilimab plus hypofractionated radiotherapy(5 Gy x 5)	NCT04636008
Corrò et al. (2022) (59)	Switzerland phase II, single-arm study	Patients with localized RC	combining pembrolizumab with short-course radiotherapy	NCT04109755
Laengle et al. (2021) (60)	Austria phase II clinical trial	Patients with LARC	neoadjuvant chemoradiotherapy in combination with ipilimumab and nivolumab	NCT04124601

LARC, locally advanced rectal cancer; RC, rectal cancer; MSI-H, microsatellite instability high; dMMR, deficient mismatch repair; TME, Total mesorectal excision.

## 5 Predictors of the efficacy of RIT

Biomarkers that can predict the clinical efficacy of RIT must be identified because only some patients benefit from this treatment.

### 5.1 PD-L1 expression

PD-L1 expression is a promising biomarker for identifying patients with CRC who may benefit from sequential radiotherapy combined with immunotherapy. In a clinical study that investigated the efficacy of preoperative CRT plus nivolumab, researchers analyzed 38 pre-CRT samples and demonstrated that the pCR rate of patients with PD-L1 proportion scores ≥1% was higher than those with PD-L1 proportion scores <1% (75% vs. 17%, *P* = 0.004) (45). Similarly, Lin et al. reported that patients with positive PD-L1 expression had a higher pCR rate (44). In addition, previous studies have shown that anti-PD-1 therapy may produce better clinical results for tumors with high PD-L1 expression than for PD-L1-negative tumors (61, 62).

### 5.2 Tumor mutational burden

Patients with high TMB have demonstrated good responses to anti-PD-1 therapy because TMB is associated with a high frequency of new antigens (63). A study of patients with MSS LARC who received combined radiotherapy and immunotherapy discovered that the TMB of patients with tumor regression grades (TRG) 0–1 was significantly higher than that of patients with TRG 2–3 (45). This finding suggests that higher TMB in pre-treatment samples of patients with MSS LARC may be a potential predictor of good outcomes for RIT.

### 5.3 Immunoscore-biopsy

Carine El Sissy et al. reported that Immunoscore-biopsy (IS_B_) which was determined based on densities of CD3^+^ and CD8^+^ T cells in rectal cancer biopsy samples could predict response to pre-CRT in patients with rectal cancer. The levels of IS_B_ were positively correlated with tumor response to neoadjuvant treatment (64). Besides, Sakti Chakrabarti et al. found that higher CD3^+^ and CD8^+^ T cell density was associated with higher objective response rate and disease control duration in patients with dMMR mCRC treated with pembrolizumab (65). These findings indicate that IS_B_ based on densities of CD3^+^ and CD8^+^ T cells might help predict and stratify patients who would benefit from RIT.

### 5.4 CD8^+^ T cell/effector regulatory T cell ratio

In the VOLTAGE study, tumor-infiltrating lymphocytes in 24 pre-CRT samples from patients with MSS LARC were analyzed using flow cytometry (45). The results showed that patients with a high CD8/eTreg ratio had a significantly higher pCR rate than those with a low CD8/eTreg ratio (78% vs. 13%). This suggests that the number of CD8^+^T cells is a positive predictor of efficacy, whereas the number of Treg is a negative predictor of efficacy. Tregs can suppress the anti-tumor immune response, and their infiltration in tumor tissues is usually associated with poor prognosis of patients (29).

### 5.5 Fibroblast growth factor receptor 1-3 deletions

Lin et al. (44) found that patients with LARC without FGFR1-3 deletions might have a better tendency for pCR when they received preoperative short-course radiotherapy followed by chemotherapy and camrelizumab. In their study, none (0/5) of the patients with FGFR1-3 deletions achieved pCR, whereas 55.6% (5/9) of the patients without FGFR1-3 deletions achieved pCR. FGFR2 promotes PD-L1 expression in CRC *in vivo* and *in vitro* via the JAK/STAT3 signaling pathway (66). Thus, the predictive significance of FGFR1-3 deletion in further large-scale studies must be explored.

## 6 Discussion

Conventional treatment options for CRC typically involve a combination of surgery, chemotherapy, and radiation, depending on disease location and progression. However, studies have shown that 54% of patients with rectal cancer experience relapse following neoadjuvant CRT combined with TME surgery (67). Approximately 66% of patients with stage II–III colon cancer and 50% of patients with stage II-III rectal cancer require adjuvant chemotherapy or CRT, respectively (68). Traditional chemotherapy can cause challenging toxic effects. Oxaliplatin-induced neuropathy and chemotherapy-related diarrhea are the most common side effects (69, 70). Other common intestinal dysfunctions include an increased defecation frequency, urinary incontinence, radiation proctitis, and perianal stimulation, which are more common in patients with rectal cancer, especially those who have received radiotherapy (71). Furthermore, nearly 50% of patients with mCRC exhibited resistance to chemotherapy based on 5-FU (72), and nearly 50% of patients with rectal cancer showed resistance to radiotherapy (73), leaving many patients with advanced CRC with limited options. Therefore, exploring more effective treatment strategies to supplement traditional CRT regimens is of great importance for patients with CRC. Immunotherapy has emerged as a promising option, and some ICIs have become the first-line treatment for mCRC. Owing to the subtle interaction between the radiation effect and the body’s immune response, the synergistic effect of radiotherapy and immunotherapy may offer new options for managing patients with CRC.

### 6.1 Limitations of current RIT clinical studies

A few clinical trials on RIT have proven effective, but limitations still exist. First, the sample size of the population included in these studies was small (most no more than 50 patients), and most trials were non-randomized single-arm phase I or phase II trials, making the representativeness of the studies relatively weak. Second, due to the late start of most studies, the follow-up time has not yet met expectations, resulting in no relevant long-term survival data, making it impossible to accurately judge the long-term benefits and adverse events. Therefore, a comprehensive evaluation of RIT based on the results of more phase III clinical trials is necessary in the future. Furthermore, multiple regimens should be used to conduct more comprehensive randomized controlled trials. Moreover, the standard CRT regimen for patients with LARC or the strategy of immunotherapy alone for patients with mCRC must be compared with RIT to better evaluate the advantages and disadvantages of RIT.

### 6.2 Exploring appropriate dosing and fractionation regimens for radiotherapy when combined with immunotherapy

The optimal radiotherapy dose and fractionation regimen to optimize the benefits of RIT remains unclear. Previous studies have demonstrated that radiotherapy induces an anti-tumor-immune effect induced by generating type I interferon (IFN) triggered through local high-dose radiation, thus initiating the innate and adaptive immune attack on tumors (74, 75). However, exposing tumor to a single high radiation dose (> 12–18 Gy) activates the deoxyribonucleic acid (DNA) exonuclease TREX1, preventing irradiated tumor cells from releasing IFN-β, and impairing the increased immunogenicity induced by radiotherapy (76). In a study of patients with MSS mCRC, Monjazeb et al. (38) found that hypofractionated radiotherapy (8 Gy × 3) or low-dose fractionated radiotherapy (0.5 Gy × 16) with the same immunotherapy agents could elicit different immune responses. Moreover, whether the optimal neoadjuvant radiotherapy regimen for patients with LARC is short-course radiotherapy (5 Gy × 5) or long-course radiotherapy (2 Gy × 25) remains debatable. Therefore, the appropriate dose and fractionation scheme of radiotherapy when combined with immunotherapy in patients with CRC requires further exploration.

### 6.3 The order of application of radiotherapy and immunotherapy and the timing of immunotherapy

In addition, the application sequence of radiotherapy and immunotherapy and immunotherapy timing are often heterogeneous. The proper sequence of RIT may depend mainly on the specific immunotherapeutic agent used and the dose or fraction of radiotherapy, as they might lead to different toxicities and clinical results. A study by Parikh et al. (40) on patients with MSS mCRC revealed that patients received immunotherapy first, followed by radiotherapy. Finally, 10% (4/40) of patients withdrew early because of immunotherapy toxicity without the opportunity to receive radiation. Therefore, further research is warranted to determine the appropriate sequence of combination therapy and immunotherapy timing.

### 6.4 Finding markers that could identify CRC groups that actually benefit from RIT

From the perspective of molecular mechanisms, RIT-related molecular biological markers other than mismatch repair genes must be identified to accurately identify CRC populations that may benefit from RIT. The relationship between radiotherapy and immunotherapy may be more profound and complex than previously thought. Studies have identified several factors that regulate radiosensitivity and IC expression, such as poly adenosine-diphosphate-ribose polymerase (PARP) inhibitors, which may induce immunosuppression by up-regulating PD-L1 expression (77), while p53, a radiation response, was confirmed to regulate PD-L1expression (78). Immunotherapy may also affect the tumor radiation response through mechanisms that are independent of its effects on immune cells. Given the lack of definitive evidence that immunotherapy may directly or indirectly sensitize radiation, more relevant preclinical and clinical studies are required to further explore the relevant mechanisms.

### 6.5 Risks of combining radiotherapy and immunotherapy

The potential of immunotherapy as a radiation sensitizer has not yet been established, and the combination of radiotherapy and immunotherapy may also be a double-edged sword. ICIs can alter the balance between immune response and immune tolerance, leading to an over-immune reaction in normal tissues. RIT may exacerbate immune-related adverse events, such as fatigue, rash, dermatosis, colitis, and gastrointestinal complications (79). In addition, radiotherapy not only promotes tumor-specific antigens recognition in tumor tissues, but also triggers the release of non-tumor-specific antigens into the tumor microenvironment. Some non-tumor-specific antigens may activate autoreactive T cells and attack normal tissues (80). Further studies are required to clarify the biological mechanisms underlying these toxic reactions and how to reduce the risk of adverse events. Overall, the ideal research methodology of clinical trials combining radiotherapy and immunotherapy are first required to preplan for the final analyses, including the addition of appropriate surrogate and intermediate endpoints. Full consideration of the exact benefits and risks of RIT should be based on increasing the enrollment of more qualified patients and decreasing the incidence of adverse events when refining the current regimens.

## 7 Conclusion

In general, this review summarized the current application of radiotherapy and immunotherapy in CRC treatment, elaborated on the underlying mechanisms of RIT, systematically reviewed the preliminary results of RIT-related clinical trials for CRC, and suggested key considerations for the future development of RIT protocols. Our study suggested that rational RIT regimens can improve the outcomes of some patients with CRC, although current studies have some limitations. Further studies on RIT should focus on including larger sample size and standardizing the combination therapy regimens based on the underlying influencing factors.

## Author contributions

JS and ZS contributed to the conception and design, acquisition and interpretation of data and drafting of the article. ZG and DH contributed to the conception and drafting of the article. HH contributed to data acquisition. JG contributed to the conception and design and revised the full text critically for important intellectual content. All authors contributed to the article and approved the submitted version.
